# Ocean mover’s distance: using optimal transport for analysing oceanographic data

**DOI:** 10.1098/rspa.2021.0875

**Published:** 2022-06

**Authors:** Sangwon Hyun, Aditya Mishra, Christopher L. Follett, Bror Jonsson, Gemma Kulk, Gael Forget, Marie-Fanny Racault, Thomas Jackson, Stephanie Dutkiewicz, Christian L. Müller, Jacob Bien

**Affiliations:** ^1^ Data Sciences and Operations, University of Southern California, California, CA, USA; ^2^ Center for Computational Mathematics, Flatiron Institute,New York, NY, USA; ^3^ Department of Earth, Atmospheric and Planetary Sciences, Massachusetts Institute of Technology, Cambridge, MA, USA; ^4^ Earth Observation Science and Applications, Plymouth Marine Laboratory, Plymouth, UK; ^5^ Department of Statistics, LMU München, Munich, Germany; ^6^ Institute of Computational Biology, Helmholtz Zentrum München, Neuherberg, Germany; ^7^ School of Environmental Sciences, University of East Anglia, Norwich, UK

**Keywords:** Wasserstein distance, earth mover’s distance, data-model comparison, optimal transport, chlorophyll, remote sensing

## Abstract

Remote sensing observations from satellites and global biogeochemical models have combined to revolutionize the study of ocean biogeochemical cycling, but comparing the two data streams to each other and across time remains challenging due to the strong spatial-temporal structuring of the ocean. Here, we show that the Wasserstein distance provides a powerful metric for harnessing these structured datasets for better marine ecosystem and climate predictions. The Wasserstein distance complements commonly used point-wise difference methods such as the root-mean-squared error, by quantifying differences in terms of spatial displacement in addition to magnitude. As a test case, we consider chlorophyll (a key indicator of phytoplankton biomass) in the northeast Pacific Ocean, obtained from model simulations, *in situ* measurements, and satellite observations. We focus on two main applications: (i) comparing model predictions with satellite observations, and (ii) temporal evolution of chlorophyll both seasonally and over longer time frames. The Wasserstein distance successfully isolates temporal and depth variability and quantifies shifts in biogeochemical province boundaries. It also exposes relevant temporal trends in satellite chlorophyll consistent with climate change predictions. Our study shows that optimal transport vectors underlying the Wasserstein distance provide a novel visualization tool for testing models and better understanding temporal dynamics in the ocean.

## 1. Introduction

Understanding the differences between large spatio-temporal datasets is a common task in oceanography. Whether quantifying the agreement between the output of an ocean simulation model [1,2] and *in situ* measurement [3,4], or monitoring the changes in the ocean across time [5], one needs a meaningful notion of ‘distance’ between scalar fields defined across the ocean. We focus on the case in which the scalar field of interest represents the density or concentration of a quantity over space. It is most common to compare images or data distributions using a ‘pixel-by-pixel’ or pointwise difference [2,6–8]; popular examples of such distances include root-mean-squared error (RMSE) and mean absolute error. However, although easy to compute, pixel-wise comparisons may not fully account for the spatio-temporal nature of ocean data, which can exhibit complicated patterns composed of both global and local underlying trends linked to shifting and evolving water mass bodies.

These issues are well known and have led to the development of various normalized differences or ‘cost functions’, which differentially weight differences arising from deviations in quantity or location, or from unresolved scales (e.g. [2,7,9]). Focusing on the probability distribution over predefined regions (e.g. marine provinces or water masses) is one way to account for spatial errors. This method has been used to examine, for example: the volumetric census of water masses [10,11]; relationships between primary production and export [12]; and the effects of mesoscale eddies [13]. Power-spectra further provide a useful basis for comparison as a function of space and/or time scale (e.g. [7,14]). Despite these advances, there remains a need for metrics that take into account pattern differences in a clear and interpretable way. This is especially true when evaluating the skill (or error) of ocean biogeochemical model simulations compared with other data sources such as satellite-derived measurements. Indeed, a recent summary paper [15] reports the need for a better measure of ocean chlorophyll difference that goes beyond pixel-wise differences. The reasons are many. Computer simulations may not be finely resolved enough to capture mesoscale chlorophyll patterns (e.g. eddies) in time and space. However, such features will be captured *in situ* and using satellites. Further, small spatial mismatches can result in large pixel-wise differences—see §5.3.2 of [15]—which penalize models that are mechanistically correct for stochastic fluctuations. What we need is a metric that is easy to interpret, like RMSE, but for pattern differences.

In this paper, we explore the use of the Wasserstein distance [16], which sometimes goes by the name earth mover’s distance [17]. As that name suggests, the Wasserstein distance measures the total amount of ‘dirt’-moving that would be required to transform one mound of dirt (representing a probability distribution) to make it equivalent to another mound (a second probability distribution). The probability distributions in our context are normalized versions of the scalar fields. Unlike pixel-by-pixel distances, the Wasserstein distance incorporates the spatial structure of discrepancies, making it particularly well suited for the comparison of ocean datasets. The Wasserstein distance has been used in several other areas of geosciences. To list a few, it has been used to analyse particle distributions in the ocean [18]; for measuring error in temperature, precipitation, and sea ice projections [19]; for ocean data assimilation [20,21]; for analysing sea height images[22]; for ocean synthetic aperture radar (SAR) segmentation [23]; and for studying sea ice imagery [24]. However, [15] makes clear that the Wasserstein distance has not been thoroughly applied to the fundamental problem of model-to-data comparison and model-skill evaluation, particularly in the context of ocean biogeochemical models and the representation of marine ecosystem structure and function. The goal of this paper is to carefully highlight the usefulness of the Wasserstein distance in this context, as well as to show its usefulness in exploring time series of satellite maps. We focus on high-coverage chlorophyll observations in the North Pacific Subtropical Gyre (NPSG) [4], and demonstrate how discrepancies between model predictions and observed chlorophyll can be interpreted in terms of a transport field that, when integrated over space, yields a measure of distance in spatial units. We do this for the comparison of surface maps (see §3(a)) and of depth profiles (see §3(b)), which reveals the long-term temporal trend and seasonality of satellite and model chlorophyll maps in §3(a)(i).

**Figure 1.**
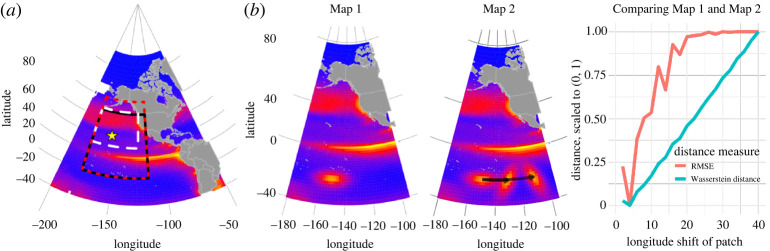
The left-most panel (*a*) shows a map of the study regions that are used for data analysis in this paper (solid lines for §3(a)(i), dotted lines for 3(a)(ii), and dashed lines for §3(a)(iii)); the star marker denotes the location of station ALOHA near Hawaii from which we obtain depth profiles of chlorophyll to analyse in §3(b). In (*b*), the first two figures show a toy example of two chlorophyll maps both formed using simulated climatology data in January (from the ocean coupled physical-biogeochemical-optical model [1,2]). One map was formed by adding an artificial patch of chlorophyll to a longitude of −150. The other map was formed by shifting this patch to the east by up to $40^{\circ }$ longitude (while also rotating it). The rightmost graph shows two different distance measures—root-mean-squared error (RMSE) and Wasserstein distance—between the two plots, while varying the amount of longitude shift of the patch. RSME plateaus after a shift of $20^{\circ }$, while the Wasserstein distance is proportional to the amount of shift. (Online version in colour.)

To convey the intuitive appeal of the Wasserstein distance over pixel-wise distance measures, consider the toy example in figure 1, in which we imagine two surface maps that are identical except for the location of an artificially inserted patch of chlorophyll south of the equator. Physical processes like, for example, Rossby waves can generate such propagating patches. The right panel shows how RMSE and Wasserstein distance quantify the difference between the two surface maps as spatial shift of the patch increases. RMSE quickly saturates: once the two patches have no spatial overlap, there is no further change in the RMSE metric. By contrast, the Wasserstein distance increases in an approximately linear fashion. Indeed, the Wasserstein distance has units of distance and is directly related to the distance that the patch has moved.

**Figure 2.**
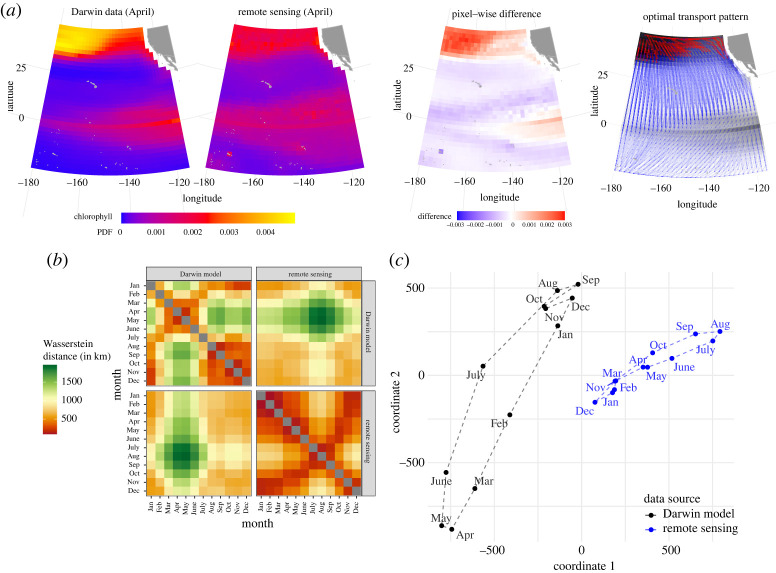
Geographical analysis of chlorophyll data. (*a*) A comparison of April’s climatology chlorophyll maps from two data sources (two left maps) using optimal transport. The first two maps are measurements on a two-dimensional grid in which each grid cell measurement can be thought of as a pixel intensity in a digital image. The values have been normalized to sum to 1 in each map. The third map in panel (*a*) shows the pixel-wise difference (which is the basis for root-mean-squared error—RMSE) of the two left maps. The rightmost map shows the estimated optimal transports (which is the basis for Wasserstein distance), with transparent blue arrows and opaque red lines showing the bottom 90% and top 10% of all the masses, respectively. This mass transfer plot shows that the major shift of chlorophyll probability mass from the concentrated upper-left corner is dispersed in a south- and eastbound direction with a particular trend. (*b*) A summary of all pairwise Wasserstein distances from the 24 maps—12 months of climatology chlorophyll maps, from the two data sources (model and remote sensing), as a 24×24 distance matrix. (*c*) A classical multidimensional scaling (MDS) performed on this data. Three notable observations can be made: (i) model data are more variable than remote sensing data, (ii) there is a clear separation between model and remote sensing data, and (iii) the connecting dashed line between adjacent months in each data source shows an annual seasonality. This is further explored in figure 3 and electronic supplementary material, figure S4, and by analysing data from each year. (Online version in colour.)

In addition to its merit as a scalar distance, the Wasserstein distance also enables the visualization of the transport that would most efficiently (from the perspective of a person moving the dirt) transform the first ocean map into the second. For example, the rightmost panel of figure 2*a* shows the optimal transport pattern between the two maps on the left (see §3(a)(i)). These optimal transport patterns are *not* to be interpreted as ‘physical’ transport of the underlying quantity. Still, these optimal transport patterns are useful for understanding *how* the data differ. In this work, we consider two primary types of comparison: (i) comparing two different data sources measuring the same signal on a spatio-temporal region or gridpoints; and (ii) comparing the same data source at different times. In both cases, visualizing the optimal transport can provide a scenario to elucidate the nature of the difference. This can be particularly useful when spatio-temporal differences are related to shifts in patterns that may not be well captured by pixel-wise comparisons.

With this paper, we aim to highlight the usefulness of studying ocean data using the Wasserstein distance, which we show is particularly well suited for evaluation of ocean biogeochemical models, among many other applications. We compare satellite chlorophyll observations from the Eastern North Pacific Ocean and depth profiles from the NPSG with their counterparts from a biogeochemical model coupled to a state estimate of the ocean currents, temperature, and salinity [2]. We show that the Wasserstein distance for chlorophyll between model and satellite data is large compared with the Wasserstein distance over the seasonal cycle from satellite data or the model. We further show how the Wasserstein distance can be used to track changes in the transitional boundaries between marine provinces over time [25]. When reduced to this ‘feature comparison’, we find that the model and satellite observations are in relatively close agreement. Furthermore, applying a similar analysis to the chlorophyll depth profiles at Station ALOHA [26,27], discrepancy between model outputs and *in situ* data is framed in terms of chlorophyll shifts along the depth dimension. Our numerical experiments allowed us to investigate whether the Wasserstein distance can effectively capture deviations in the ‘deep chlorophyll maximum’ (DCM) between two chlorophyll depth profiles [28–30]. These results provide a path and justification for using the Wasserstein distance to analyse deviations in terms of pattern displacements, and provide complementary information on magnitude differences.

## 2 Material and methods

### (a) Wasserstein distance

Consider two discrete probability distributions $P={\left(P_{i}\right)}_{i=1}^{m},Q={\left(Q_{j}\right)}_{j=1}^{n}$, such that $P_{i}\geq 0$ for all i, $Q_{j}\geq 0$ for all j, and $\sum_{i}P_{i}=\sum_{j}Q_{j}=1$. In our context, i=1,…,m indexes a spatial partition of the region of ocean being studied into m cells (and likewise for j=1,…,n) and $P_{i}$ gives the proportion of the chlorophyll (or any other positive quantity the scalar field is representing) in the region that is in cell i. In the special case that i and j index the same set of cells (such as m=n pixels), one can define pixel-wise distances such as the $\text{RMSE}(P,Q)={\left((1/n)\sum_{i}{\left(P_{i}-Q_{i}\right)}^{2}\right)}^{1/2}$. If P and Q do not exist on the same coordinates, they need to be reconciled (processed) to exist on the exact same cells in order to calculate RMSE. This requirement is not shared by the Wasserstein distance, which we describe next.

The Wasserstein distance, which is also sometimes called earth mover’s distance [17] as discussed in the introduction, can be thought of as the total amount of ‘dirt’-moving required to transform a mound shaped like P to a mound shaped like Q when one performs *optimal transport* [16,31,32], i.e. when one does this earth moving in the most efficient fashion possible. More precisely, the optimal transport between P and Q can be expressed as solving the following linear program:

2.1 $$\hat{f}=\underset{f}{\text{argmin}}\sum_{i=1}^{m}\sum_{j=1}^{n}f_{i,j}d_{i,j}^{2},\;\text{subject to }\left\{ \begin{array}{l} \forall i,j:f_{i,j}\geq 0\\ \forall i:\sum_{j}f_{i,j}=P_{i}\\ \forall j:\sum_{i}f_{i,j}=Q_{j}, \end{array}\right.$$

where $d_{i,j}$ is the *base distance* between cell i in P and cell j in Q. The optimization variable $f_{i,j}$ describes the amount of probability mass being transported from i to j. The constraints encode that no mass is created or destroyed and that the net effect of the transport is to take P to Q. The objective function is a weighted sum of squared distances (the square used in this paper makes this the ‘2-Wasserstein’ distance), where the weights are given by the amount of probability mass being transported across all pairs of cells, i and j. The optimum $\hat{f}$ is the optimal transport between P and Q, and the Wasserstein distance is defined to be the square root of the optimal value of this optimization problem: $W_{2}(P,Q)={\left(\sum_{i=1}^{m}\sum_{j=1}^{n}{\hat{f}}_{i,j}d_{i,j}^{2}\right)}^{1/2}$.

Throughout, we use the transport R package [33], which implements the algorithm in [34] in which each discrete probability distribution first undergoes a multiscale transformation and is decomposed into a weighted sum of Gaussian bases; then the optimal transport problem is solved using a network simplex algorithm. This has O(nm) computational complexity. Solving the optimal transport problem with a full dense $d_{i,j}$ (base distance matrix as in equation (2.1)) is prohibitively slow at moderate problem sizes like n=m=10 000. One interesting and straightforward future improvement is to reduce the number of transports needed by setting $d_{i,j}=\infty$ if |i−j|>c for some threshold c. Generally, there is a large literature on algorithms to calculate optimal transport, of which we cite only a recent few. Among popular cutting-edge algorithms are fast approximations in the Fourier space [35] and in the wavelet space [36]. Also popular is entropic regularization [37], which is known as Sinkhorn distance. The most analogous pre-existing application of the Wasserstein distance is to digital image data, which has gained popularity in recent years in the neural network literature [17].

A distinctive feature of ocean applications (as opposed to, for example, digital image applications) is that the base distance $d_{i,j}$ cannot be taken to be Euclidean distance, especially when the coordinates of the cells i and j are far apart. Instead, we take the base distance to be the *great circle* distance between the (longitude, latitude) coordinates, which we compute using the geodist package in R [38]. Our work also offers fully reproducible code, via an R package named omd (https://github.com/sangwon-hyun/omd), which could be used for other ocean studies.

### (b) Multidimensional scaling

In our analysis, multidimensional scaling (MDS) plots will be used to help us interpret distance matrices, often highlighting seasonality and other relationships across time. Using the Wasserstein distance as described in §2(a), we can take a collection of maps and form a distance matrix $D\in R^{N\times N}$, where $D_{ab}$ is the Wasserstein distance between normalized chlorophyll maps a and b. To help interpret the resulting distance matrix, we visualize the maps’ relationship to each other using classical MDS [39,40]. This popular data analysis technique seeks a configuration of points in the two-dimensional plane where Euclidean distances are close to those in an inputted distance matrix. That is, after computing the Wasserstein distance between all pairs of N maps, the goal is to find a low-dimensional embedding, $z_{1},\ldots ,z_{N}\in R^{2}$, for which $||z_{a}-z_{b}|{|}_{2}\approx D_{ab}$ for all maps 1≤a<b≤N. An approximate closed-form solution can be calculated using an eigen-decomposition of the doubly centred matrix of squared distances. The details are provided in electronic supplementary material, §1.1.

### (c) Data

The analysis is based on monthly chlorophyll data from three different data sources: derived from ocean-colour remote sensing observations, the output from a global biogeochemical circulation model, and integrated *in situ* observations. We use a subdomain of the model and remote sensing datasets focused on a latitude–longitude rectangle in the Pacific Ocean directly above—and including—Hawaii. The region is centred around about $20^{\circ }$ latitude and $-155^{\circ }$ longitude and captures interesting geographic variability in the ocean. To the south of this region is the NPSG (low latitude, dominated by warm, more saline water) and to the north is the Subpolar Gyre (high latitude, low-temperature, low-salinity, nutrient-rich water). The region between these two gyres is the North Pacific Transition Zone (NPTZ) with a strong gradient in chlorophyll, as can be seen in the remote sensing observations and in the model output (figure 2*a*, left panels). We also focus on data directly from a fixed location near the south of this region, Station ALOHA ($22.75^{\circ }$ latitude and $-158^{\circ }$ longitude) [41]. Throughout, we exclude chlorophyll data near the coastline where both satellite measurements and numerical models have known irregularities. Each dataset is described in some detail next.

#### (i) CBIOMES-global model output

Model data are based on output from a coupled physical-biogeochemical-optical model, modified for the Simons Collaboration on Computational Biogeochemical Modeling of Marine Ecosystems (CBIOMES) project. The CBIOMES-global model simulates the period from 1992 to 2011 [42].

The model’s physical component is derived from the Estimating the Circulation and Climate of the Ocean project, v. 4 (ECCOv4) [2,7,8]. ECCOv4 uses a ‘least-squares with Lagrangian multipliers’ method to get internal model parameters, and initial and boundary conditions, which minimize the discrepancy between global observational data streams of satellite and *in situ* data. The end product is a global three-dimensional configuration state estimate, at a horizontal resolution of $1^{\circ }$ and with depth ranging from 10 m at the surface to 500 m at depth (see [2] for details).

The biogeochemical/ecosystem component is from the MIT Darwin Project and follows that of [43]. The model data we use in this paper is the aggregated chlorophyll-a across all phytoplankton groups simulated from this ecosystem model, made into monthly averages. The amount of chlorophyll in each of the 35 phytoplankton types varies based on light, nutrients, and temperature [44]. The 35 phytoplankton types are from from several biogeochemical functional groups such as pico-phytoplankton, silicifying diatoms, calcifying coccolithophores, mixotrophs that photosynthesize and graze, and nitrogen-fixing diazotrophs, with sizes that span from 0.6 to 228 μm equivalent spherical diameter (ESD). The model incorporates various interactions with chemical factors (e.g. carbon, phosphorus, nitrogen, silica, iron, oxygen) and with other species (e.g. grazing by zooplankton). See [43] for full details. Hereon, we will simply refer to this data as model data.

#### (ii) Remote sensing data

Remote sensing (or satellite-derived) data are based on v. 3.0 of the European Space Agency Ocean Colour Climate Change Initiative (OC-CCI) [45–47], a blended level-4 chlorophyll product with a spatial resolution of 4 km. The OC-CCI v. 5.0 combines data from five independent ocean-colour sensors to produce merged, climate-quality observations of chlorophyll concentration. The sensors include the Sea-viewing Wide-Field-of-view Sensor (SeaWiFS), the Aqua MOderate-resolution Imaging Spectroradiometer (MODIS-Aqua), the MEdium spectralResolution Imaging Spectrometer (MERIS), the Suomo-NPP Visible InfraredImaging Radiometer Suite (NPP-VIIRS), and the Sentinel 3A Ocean and Land Colour Instrument (OLCI). These data sources are algorithmically merged and processed (see more details of this processing in [4,46]), then downscaled to the same spatial grid as model data at the monthly time resolution.

#### (iii) *In situ* data from station ALOHA

We additionally consider shipboard-measured chlorophyll-a from Station ALOHA ($22^{\circ }$45${\;}^{\prime }\;N$, $158^{\circ }$00${\;}^{\prime }\;W$). The dataset (obtained from the Simons Collaborative Marine Atlas Project (CMAP), originally sourced from https://hahana.soest.hawaii.edu/hot/dataaccess.html) contains concentrations of chlorophyll collected using a CTD fluorescence sensor. There are 28 583 observations measured between 3 October 1988 and 27 November 2016 in the depth range between 0 and 200 m. This data were downloaded directly from [48], an R package for accessing the CMAP database.

In §3(b), we compare *depth profiles* (measurements over depth) of *in situ* data and model data using the Wasserstein distance. *In situ* data are sampled irregularly in time, while Darwin data is complete in space and time. In order to compile the two datasets at matching locations in space and time, we *colocalize* the model data, by taking averages of the chlorophyll measurements in a certain space-time vicinity (±2 days and ±5 m) of each time point of the *in situ* data. Figure 6*b* shows the chlorophyll data from the two sources. Each depth profile is normalized by dividing by the total so that the sum is 1 prior to calculating the Wasserstein distance, as done for the maps.

## 3. Results

### (a) Geographical and temporal analysis of chlorophyll data

In this section, we show several different data applications of the Wasserstein distance to the ocean setting, each highlighting a different aspect of ocean data comparisons. First, in §3(a)(i), we consider the climatological seasonal changes in chlorophyll patterns in both satellite and model data, and we also perform direct model–satellite comparisons. Here, ‘climatological’ refers to being based on the 12 average monthly chlorophyll levels (averaging from 1998 to 2006). Next, in §(ii), we consider the full time series of monthly averages from 1998 to 2006 and focus on using the Wasserstein distance to explore changes in chlorophyll patterns over that time period. Finally, in §(iii), we use a smaller longitude–latitude rectangle in the NPTZ, and base comparisons on estimated boundaries between regions instead of on the original chlorophyll concentrations.

#### (i) Climatology chlorophyll data

Our first comparison is between the two climatology data sources—remote sensing and model data. The third panel in figure 2*a* shows the pixel-wise difference, and portrays both large positive deviations in the northern region and smaller ones in a wider region near the equator. The right-most panel shows an example of the optimal transport pattern from comparing climatology remote sensing data and model data in April. Optimal transport is visualized as blue transparent arrows, and those corresponding to the top 10% are highlighted in bold red. Both plots indicate that the model and remote sensing data differ the most in the northern region, while optimal transport additionally shows a southbound shift in patterns across the whole domain.

Next, we form a 24 × 24 distance matrix $D={\left(D_{a,b}\right)}_{a,b}$, shown in figure 2*b*, from the (242) unique pairwise Wasserstein distances between chlorophyll maps a and b (ranging over all 12 months and both data sources). This shows interesting seasonal changes in chlorophyll patterns within each of the data sources. For instance, the Wasserstein distances in a given row (or column) in the top-left panel (model) or bottom-right panel (satellite) form a unimodal curve when plotted as a one-dimensional time series. Also, the Wasserstein distances between monthly remote sensing data in the top-left quadrant have much larger values than the Wasserstein distances between monthly model data in the bottom-right quadrant, meaning that patterns of chlorophyll shift geographically more in the Darwin model compared with the remote sensing data. The 12 Wasserstein distances between the two sources in each calendar month are shown in the diagonal values of the upper-right and lower-left quadrants and have large values compared with (i) the distances between any two months and (ii) the distances between adjacent months in either data source.

We further summarize the distance matrix D with a classical MDS plot (figure 2*c*), projecting the 24 chlorophyll maps onto a two-dimensional plot. This MDS plot again shows that model data has higher variability than the remote sensing data. It also shows a clear separation between the two data sources. The line connecting the data sources shows a closed loop within each source, which shows seasonality according to the time of year. A careful look reveals that the seasonality pattern is different for the two data sources—the distance between the three months (August through October) and (December through January) is smaller in the model data than in the remote sensing data.

#### (ii) Interannual variability and long-term trends

**Figure 3.**
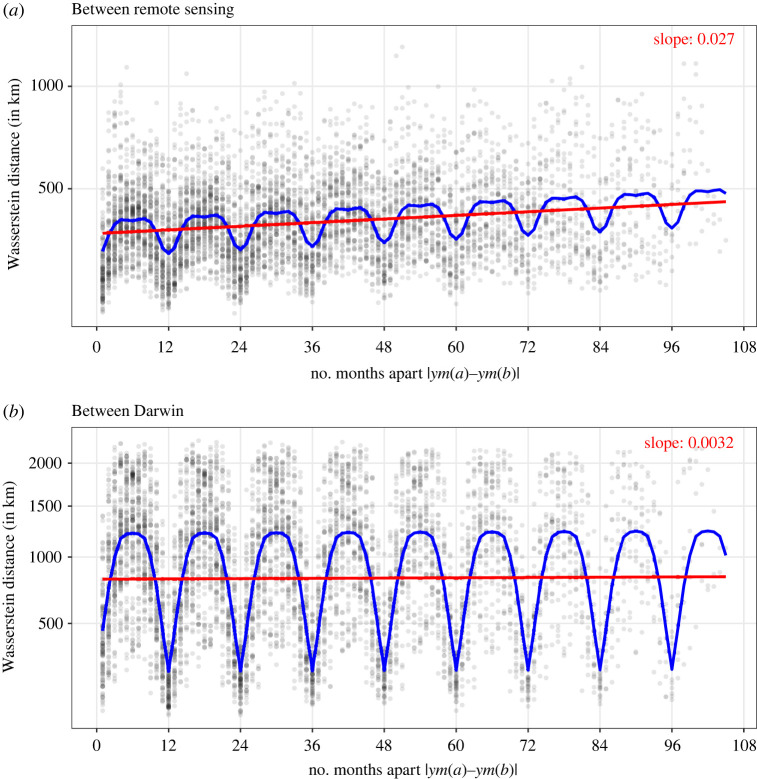
Wasserstein distance between time-resolved chlorophyll data from different months (between March 1998 and December 2006) for the (*a*) remote sensing data and (*b*) model data, arranged so that the x-axis shows how many months apart the two chlorophyll maps are and the y-axis is the Wasserstein distance (which uses square-root scaling). The blue line is fitted using a regression model that assumes a linear trend together with a regular seasonal pattern, and the red line shows the linear trend excluding the seasonal component. The slope of the red line for the remote sensing data is roughly 8.4 times larger than for the model data—both slope values are shown in the top-right of each panel. Note, the red line is linear in $\sqrt{D_{ab}}$ and only appears linear here because the slope coefficient is very small in size. (Online version in colour.)

We expand the analysis by using time-resolved data based on monthly averages of model and remote sensing data in all months available from 1998 to 2006. An MDS analysis leads to similar conclusions as those from the climatology data (see electronic supplementary material, §1.2, for a detailed analysis). Next, figure 3 plots the Wasserstein distance between pairs of maps from within a single source (model or remote sensing) as a function of the number of months they are apart. The blue line shows a regression mean that explicitly models annual seasonality, and the red line is the linear trend without the seasonality. The regression model predicts $\sqrt{D_{ab}}$ between year-month a and b, using two types of predictors: (i) the number of months apart |ym(a)−ym(b)| that includes year information and (ii) the number of *calendar months* apart if one ignores the years, i.e. |m(a)−m(b)|∈{0,…,6}. The predictor in (ii) is an explicit accounting for differences in the time of year. In particular, the fitted model for $\sqrt{D_{ab}}$ shown by the blue line is given by

3.1 $${\hat{\beta }}_{0}+{\hat{\beta }}_{1}\cdot |ym(a)-ym(b)|+\sum_{k=0}^{6}{\hat{\beta }}_{2,k}1(|m(a)-m(b)|=k),$$

where $\sum_{k=0}^{6}{\hat{\beta }}_{2,k}=0$. The red line is simply the first two terms of the above expression. The undulating blue line indicates the larger seasonal variability in chlorophyll patterns in the model relative to remote sensing data noted in §3(a)(i). The slope of the red line, ${\hat{\beta }}_{1}$, is positive for remote sensing and 8.5 times that of the model data. Indeed, the upward trend of the red line for the remote sensing data is visibly much more apparent than that for the model data. This suggests that the chlorophyll maps in the remote sensing data are getting increasingly more different from each other (i.e. there is a trend in the chlorophyll patterns) in a way that is not reflected in the model. This is further supported by electronic supplementary material, figure S5, which shows a sustained trend in the remote sensing data over a longer time period (1996–2020), as well as by the MDS plots in electronic supplementary material, figure S6. Using RMSE instead of Wasserstein distance in electronic supplementary material, figure S7, the increasing trend is weaker but still present, and about two times larger in remote sensing data than in model data.

**Figure 4.**
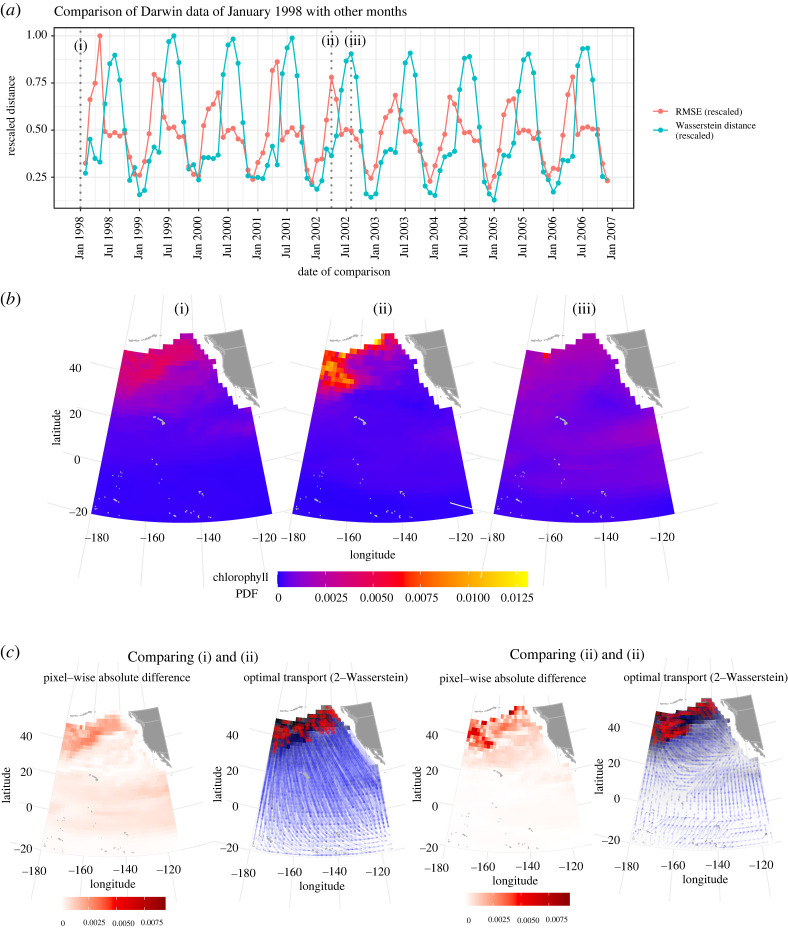
Comparison between the interpretation of time series data using Wasserstein distance and root-mean-squared error (RMSE). (*a*) The distance between January 1998 model data (i) to other months’ model data, measured by Wasserstein distance and RMSE, with distances normalized to range from 0 to 1. (*b*) The three maps. Noticing that the seasonal cycle and annual peak of Wasserstein distance is different in the two sources, we focused on two months—April 2002 (ii) when RMSE peaks and August 2002 (iii) when Wasserstein distance peaks. (Grey vertical dotted lines are drawn at dates (i), (ii), and (iii) for emphasis.) RMSE measures January to be more different from April than it is from August, while Wasserstein distance measured the opposite. In (*c*), the optimal transport between (i) and (ii) is mostly short shifts locally in the north, while the pixel-wise difference is overly pronounced due to a few large differences in the northern coastal region. On the other hand, the optimal transport between (i) and (iii) includes two types of shifts—those that are local to the northern region, and sizeable equator-bound shifts. The pixel-wise difference does not capture the latter. Note, only half of the arrows are shown in the optimal transport plots for visual clarity. The year of 2002 was chosen randomly, and the same analysis using another comparison year shows similar conclusions. (Online version in colour.)

Lastly, figure 4 highlights a stark contrast between Wasserstein distance and RMSE. The lines plotted in panel *a* show the distance from model data in January 1998 to all other months of model data in our date range, measured in two ways (Wasserstein distance and RMSE). Both have regular seasonality, but the Wasserstein distance curve peaks in the summer (around August) of each year, while the RMSE curve peaks in the early spring (around April). We focus on three months—shown as January 1998 (I), April 2002 (II), and August 2002 (III) in panel *a*—and note that the domain of calculations has been extended further northward as compared with figure 2.

In panel *b* comparing (I) and (II), we see that the RMSE is relatively high due to a few large mismatches in the coastal region, while the Wasserstein distance in this comparison is relatively small because only local shifts exist in the north. On the other hand, panel *c* comparing (I) and (II) shows that the Wasserstein distance is appropriately large; the rightmost figure shows how optimal transport captures many global southbound shifts in probability mass to the equatorial region. Pixel-wise difference (third figure from the left) fails to capture this visibly large pattern difference, and RMSE is measured to be smaller than from the comparison in panel *b*. This demonstrates how the Wasserstein distance can be an improvement over RMSE in quantifying such differences between maps.

#### (iii) Comparing ocean provinces

Sometimes, rather than comparing the scalar fields directly, we may be more interested in comparing a scientifically relevant derived feature of the fields. For example, one may algorithmically segment the ocean into cohesive regions—‘provinces’—based on underlying differences in one or more fields (e.g. [49–52].

**Figure 5.**
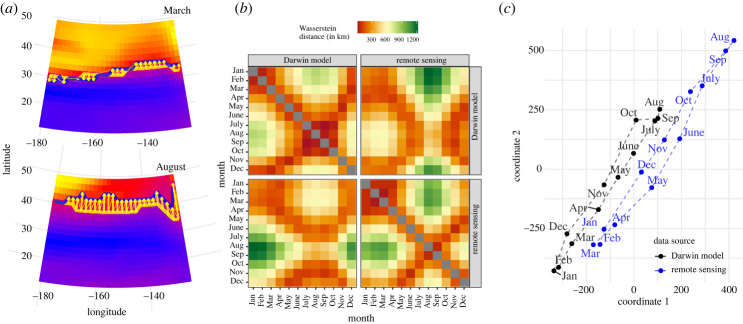
Comparison of ocean provinces using Wasserstein distance ($W_{2}$). (*a*) An example of the application of Wasserstein distance on cluster boundaries for March and August based on chlorophyll climatology data (the full set of plots from all months is provided in electronic supplementary material, figure S8). The plots show province boundaries estimated from remote sensing (blue line) and model (yellow line) data, overlaid on model chlorophyll data shown as heatmaps. The next two panels show summaries of all pairwise Wasserstein distances from the 24 maps of estimated cluster boundaries (for the 12 months of climatology chlorophyll maps from the two sources) in the same style as figure 2. (*b*) A 24×24 distance matrix and (*c*) panel show a classical multidimensional scaling (MDS) performed on this data. The distance between the two data sources in the same month is small and the seasonal dynamic shown by the lines is similar in the two data sources. This shows that, despite the large between-source distance between chlorophyll maps in figure 2, one important aspect—the estimated boundary between the two bodies of water (the North Pacific Transition Zone and the Subtropical Gyre)—is similar between the two data sources. (Online version in colour.)

We show here how the Wasserstein distance can be used to evaluate how different the boundaries are of such provinces when determined from different datasets or algorithms. Here, we apply a clustering algorithm (K-means clustering) to two chlorophyll maps—one from remote sensing and the other from the model—to estimate two different spatial provinces of chlorophyll. In our study region, this province boundary occurs in the NPTZ and is often referred to as the Transition Zone chlorophyll Front (TZCF) [25,53]. We demonstrate in this section how to use the Wasserstein distance to flexibly measure the difference between ocean provinces, by measuring how much transport is needed to move the boundaries of one set of provinces (based on model data) to make them equivalent to that of an alternative definition of provinces (based on remote sensing data). Given a partition of the ocean, we can extract a binary scalar field that is 0 inside the provinces and equal to a non-zero constant along the discretized boundaries between regions. Given two such binary scalar fields, we can then apply the Wasserstein distance. An example is shown in figure 5*a* for the March and August chlorophyll climatologies, where the estimated boundary is shown as yellow (model) and blue (remote sensing) lines.

**Figure 6.**
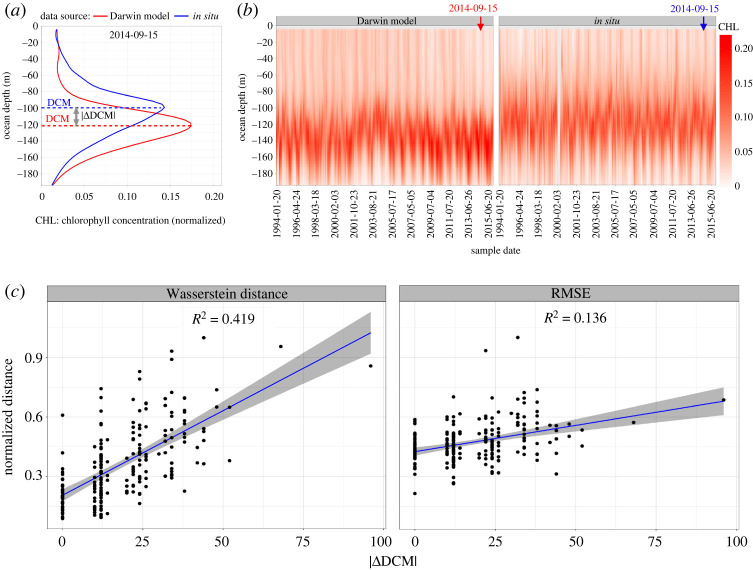
Comparing depth profiles of chlorophyll from two data sources. (*a*,*b*) Depth profiles of chlorophyll from two data sources—model and *in situ*—with an example of a single depth profile for 15 September 2013 given in (*a*) and all depth profiles between October 1988 and November 2016 (n=226) given in (*b*). Each vertical slice (a single one-dimensional histogram of chlorophyll distribution from each data source) at overlapping time points can be compared using Wasserstein distance ($W_{2}$).(*c*) The effectiveness of the two distance measures, root-mean-square error (RMSE) and Wasserstein distance (y-axis), in capturing the difference in the deep chlorophyll maximum (DCM, x-axis) recorded from the model and at Station ALOHA (*in situ*) at shared dates. DCM refers to the region below the ocean surface where the maximum concentration of chlorophyll is observed. The higher $R^{2}$ demonstrates that Wasserstein distance is better able to capture the variability in the difference in the DCM than RMSE. (Online version in colour.)

It is interesting to compare the distance matrices (panels *b*) and the MDS plots (panels *c*) in figure 5 and figure 2, which were formed by applying the Wasserstein distance to the chlorophyll field itself. When performing the Wasserstein distance on the boundaries, the MDS plot in figure 5 shows little between-source difference (compared with within-source seasonal variability), with the months from the two data sources lining up with each other. By contrast, the MDS plot of figure 2 shows a larger degree of between-source variability. In other words, despite the relatively large between-source distance between chlorophyll maps, we see that in terms of one important aspect—the estimated boundary between the regions—the two data sources agree rather well. Putting this in the context of data source comparison, boundary comparison shows a much better connection between the model and remote sensing data than the chlorophyll fields themselves, suggesting the model captures the overarching patterns and controls although not the exact locations and more detailed patterns.

### (b) Comparing depth profiles of chlorophyll

In this section, we use the Wasserstein distance to compare chlorophyll depth profiles at Station ALOHA using two different data sources (*in situ* and model). In the vertical profile of chlorophyll, a DCM (sometimes also referred to as a subsurface chlorophyll maximum, SCM [54]) is observed as a pronounced peak at depth (generally below the first optical depth) (figure 6). A DCM develops under stratified conditions [55] at the point of cross-over between two conditions that limit phytoplankton growth. Surface waters are light-rich and nutrient-limited, while at depth, nutrient concentrations are high and photosynthesis is light-limited [56,57]. At the depth of cross-over between these conditions, a DCM can develop [58–60] and the consumption of nutrients by phytoplankton acts to fix this DCM at a given depth.

Figure 6 shows Wasserstein distance and RMSE comparisons between chlorophyll depth profiles from two data sources—*in situ* and model—at 226 shared dates between October 1988 and November 2016. Panel (*a*) shows an example of a single chlorophyll depth profile for the two data sources (for 15 September 2014), while all 226 depth profiles for each data source are shown in (*b*). For each comparison (i.e. each common date), we also record an estimate of the DCM, measured by the depth at which the maximum concentration of chlorophyll occurs. Panel (*c*) shows linear regressions of Wasserstein distance and RMSE on the estimated difference in DCM between the two data sources. The higher $R^{2}$ of the left panel of figure 6*c* suggests that Wasserstein distance is more effective than RMSE at capturing the observed difference in DCM. Additionally, electronic supplementary material, figure S9, shows that the most prominent movement across depth—pooled across all comparisons made—is from approximately 96 m in the *in situ* data, to 140 m in the model data. This indicates that in aggregate, there is a depth-wise mismatch in the DCM between the two data sources. The Wasserstein distance uncovers the spatial mismatch without the additional step of isolating the DCM.

## 4. Conclusion

We have demonstrated through a series of examples how the Wasserstein distance can be a useful tool for oceanographers performing the common task of comparing scalar fields in the ocean. Our analyses focused on two time-varying chlorophyll datasets in the Pacific Ocean—a map defined over a longitude–latitude box in the North Pacific and a depth profile at Station ALOHA. In several examples, we found that the Wasserstein distance was able to capture differences in seasonality, distribution shifts, and other scientifically relevant factors in ways that a pixel-wise difference could not. For example, in the depth profile analysis, the Wasserstein distance could more closely track the changes in the DCM than RMSE. A further advantage over RMSE that we did not demonstrate in our examples is that the Wasserstein distance does not require the two sources to be defined on identical sets of spatial cells.

Our Wasserstein distance-based analysis also suggested that the differences in chlorophyll data from the model and remote sensing observations can sometimes be larger than the within-source seasonal variability. The optimal transport maps that are generated in the computation of Wasserstein distance allowed us to understand that this difference was driven by a seasonally varying set of global-scale probability mass shifts. We also found that a key feature of these two data sources—the estimated boundary between the subpolar zone and the subtropical gyre—are much more similar in this region than the original chlorophyll maps. Analysis of the Wasserstein distance on remote sensing data (further analysed with a linear regression with customized covariates) also helped reveal a long-term change from 1998 to 2006 that is not present in the model data. This suggests the usefulness of the Wasserstein distance for examining spatial data over time within a single source. Current studies often establish long-term trend terms of changes in magnitude; the Wasserstein distance detects changes in *patterns*, which may help detect long-term trends efficiently and with less uncertainty.

The demonstrations within this paper are just a starting point for the potential uses of the Wasserstein distance. We envisage this metric being used by many oceanographic data scientists for a variety of comparisons, across a range of dimensions and variables. One particular future development of interest would build on our application of Wasserstein distance to province boundaries with exploration of this technique for more complex applications than the single horizontal TZCF boundary demonstrated here. Defining and testing provinces (biomes) in the ocean is an active area of research [51,52], and we believe that the Wasserstein distances can provide a flexible tool to compare competing definitions of biomes.

As demonstrated in our examples, the Wasserstein distance is particularly useful for model-data comparison because models can struggle to get the physical location of some key features in the ocean, such as the Gulf Stream. A pixel-wise comparison will measure the magnitude of difference at rigid locations, while the Wasserstein distance will focus on the pattern change and appropriately measure this discrepancy in the longitude–latitude space.

Further, the regression analysis in §3(a)(ii) suggests the Wasserstein distance as a powerful tool to examine *temporal* trends in patterns rather than in magnitudes. This shows the Wasserstein distance goes far beyond simple model-data comparison, and can be useful for analysing spatial fields of ocean physical, biogeochemical, and optical quantities over time.

Developing computational improvements will be important to allow for full global ocean comparisons. One simple extension is to only allow local transports, by directly modifying the base distances. Handling this sparser structured base distance effectively—by building specialized software—may be an important practicality. Faster approximations to optimal transport are popular in computer science and machine-learning applications, and can also be adopted when analysing ocean data.

Another methodological extension is to consider optimal transport with unequal masses [61]; a natural scenario when dealing with physical quantities in the ocean. Normalizing such data prior to analysis discards a potentially important piece of information, which is the total amount of mass prior to normalization. When the data in a few bins are very large, the normalization can unduly flatten the probability mass in other bins. An interesting future direction is to allow optimal transport to borrow from physical transport to become more physically realistic. Optimal transport is not to be confused with physical transport of the underlying quantity in the ocean. Instead, optimal transport can be thought of as an alternative measure of distance that measures pattern shifts in the space of the data. Nonetheless, making the optimal transport more physically constrained could be a beneficial future direction. To do so, one could adjust the base distance $d_{ij}$ to account for factors such as natural boundaries in the ocean (e.g. two clear bodies of water that do not mix), or ocean currents that prevent or promote movement in certain directions. For example, by simulating Lagrangian drifts of particles under known currents, one might be able to form a more oceanographically relevant base distance that is then inputted into the Wasserstein distance calculation.

## Data Availability

Data and code are available at https://github.com/sangwon-hyun/omd. All data used in this article is already available to the public via the UCI Machine Learning Respository. We have submitted the code used to generate the graphs and tables in the paper as electronic supplementary material [62] and grant permission for this to be made public.
